# Connectivity Loss in Experimental Pond Networks Leads to Biodiversity Loss in Microbial Metacommunities

**DOI:** 10.1111/gcb.70001

**Published:** 2024-12-13

**Authors:** Beáta Szabó, Máté Váczy‐Földi, Csaba F. Vad, Károly Pálffy, Thu‐Hương Huỳnh, Péter Dobosy, Ádám Fierpasz, Zsuzsanna Márton, Tamás Felföldi, Zsófia Horváth

**Affiliations:** ^1^ Institute of Aquatic Ecology HUN‐REN Centre for Ecological Research Budapest Hungary; ^2^ National Multidisciplinary Laboratory for Climate Change HUN‐REN Centre for Ecological Research Budapest Hungary; ^3^ Department of Pharmacology and Pharmacotherapy, Faculty of Medicine Semmelweis University Budapest Hungary; ^4^ Doctoral School of Biology, Institute of Biology ELTE Eötvös Loránd University Budapest Hungary; ^5^ Department of Microbiology ELTE Eötvös Loránd University Budapest Hungary; ^6^ Department of Plant Systematics, Ecology and Theoretical Biology ELTE Eötvös Loránd University Budapest Hungary

**Keywords:** aquatic microorganisms, biodiversity loss, connectivity loss, dispersal, habitat fragmentation, mesocosm

## Abstract

Habitat fragmentation is among the most important global threats to biodiversity; however, the direct effects of its components including connectivity loss are largely unknown and still mostly inferred based on indirect evidence. Our understanding of these drivers is especially limited in microbial communities. Here, by conducting a 4‐month outdoor experiment with artificial pond (mesocosm) metacommunities, we studied the effects of connectivity loss on planktonic microorganisms, primarily focusing on pro‐ and microeukaryotes. Connectivity loss was simulated by stopping the dispersal among local habitats after an initial period with dispersal. Keeping the habitat amount constant and the abiotic environment homogeneous allowed us to track the direct effects of the process of connectivity loss. We found that connectivity loss led to higher levels of extinction and a decrease in both local and regional diversity in microeukaryotes. In contrast, diversity patterns of prokaryotes remained largely unaffected, with some indications of extinction debt. Connectivity loss also led to lower evenness in microeukaryotes, likely through changes in biotic interactions with zooplankton grazers. Our results imply that connectivity loss can directly translate into species losses in communities and highlight the importance of conserving habitat networks with sufficient dispersal among local habitats.

## Graphical abstract

In an outdoor mesocosm experiment, we studied the effects of habitat‐network fragmentation (i.e., the loss of connectivity between habitats) on planktonic prokaryote and microeukaryote communities. Connectivity loss resulted in a decrease of both local and regional diversity and reduced the survival probability in microeukaryotes, while prokaryotes remained largely unaffected. In addition, connectivity loss indirectly contributed to decreasing microeukaryote evenness via modulating biotic interactions with zooplankton grazers.

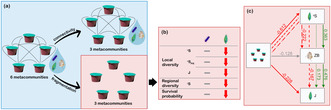

## 1 Introduction

Habitat loss is one of the primary drivers of global biodiversity decline (Brooks et al. 2002; Hanski 2011; Pimm 2008; WWF 2018) that can be manifested as two major mechanisms. First, it affects habitat networks directly when species disappear due to the sampling effect related to the species–area relationship (Fahrig 2013). At the same time, the spatial configuration of the remaining habitats may also undergo changes (Fahrig 2017), which involves both the fragmentation of large areas and an increased spatial isolation through the loss of connectivity between habitat patches (Haddad et al. 2015; Horváth et al. 2019). In addition, functional connectivity loss can occur without a significant reduction in total habitat amount via decreasing landscape permeability driven by increasing urbanization and land use changes (Oertli and Parris 2019; Trombulak and Frissell 2000). While these processes can all have a negative impact on biodiversity, their relative roles still represent a highly debated topic in ecology (Fahrig 2019; Fletcher et al. 2018; Valente et al. 2023). This largely stems from a general lack of direct evidence on the role of connectivity loss. With only a few exceptions (e.g., Gonzalez et al. 1998; Graham et al. 2022; Haddad et al. 2017; Horváth et al. 2019), most studies have so far inferred its consequences for biodiversity based on observed connectivity gradients rather than tracking biodiversity changes following actual connectivity reductions over time.

Connectivity loss can have several negative impacts on biodiversity. First, it can lead to increased probabilities of local extinction by directly decreasing colonization rates (Fahrig 2002), the exchange of genes and individuals, and contributing to inbreeding (Frankham 2006; Hagen et al. 2012; Harrisson et al. 2012; Klinga et al. 2019) and ecological drift resulting from demographic stochasticity (Chase et al. 2020; Ryberg, Smith, and Chase 2012). In addition, it can enhance the importance of further stochastic processes, such as priority effects (Chase 2003; Woody et al. 2007). Connectivity loss can also indirectly modify density‐dependent biotic processes (Hagen et al. 2012; Magrach et al. 2014; Peh et al. 2014), resulting in changes in community structure and richness (Berga et al. 2015; Wardle 2016; Wardle and Zackrisson 2005), or functional diversity (Wardle 2016; Wardle and Zackrisson 2005), ultimately reflected in altered ecosystem functioning (Gonzalez, Rayfield, and Lindo 2011). Additionally, it can decrease the stability and persistence of populations during environmental change (Hassell, Godfray, and Comins 1993; McCann, Rasmussen, and Umbanhowar 2005). On the other hand, fragmentation might also have a stabilizing effect on unstable resource–consumer relationships via rescue effects and spatial refuges (Briggs and Hoopes 2004). Moreover, it can reduce the effect of predation, spread of disturbances (Levin et al. 2012) and diseases (Gonzalez et al. 1998; Graham et al. 2022; Haddad et al. 2017; Hess 1994). This overall highlights the importance of considering biotic interactions as underlying mechanisms of fragmentation effects, further contributing to the challenges of accurately forecasting its consequences.

Studies on the effects of connectivity loss on biodiversity have so far mostly focused on continuous habitats undergoing fragmentation, mainly in terrestrial biomes (e.g., Gonzalez et al. 1998; Graham et al. 2022; Haddad et al. 2017). Consequently, our understanding of how connectivity loss affects biodiversity in naturally patchy, insular types of ecosystems, such as small standing waters, remains poorly known. Micro‐ and mesocosm experiments can address these limitations as under experimental conditions, it is more feasible to disentangle the direct effect of connectivity loss from other factors, such as habitat size and amount, local environmental variables, or interspecific interactions.

Studies in fragmented landscapes have traditionally focused more on macroorganisms (e.g., Branco et al. 2012; Hamer 2016; Hamer, Mechura, and Puky 2023; Johnson et al. 2013; Rösch et al. 2013), while there is a clear knowledge gap with especially scarce experimental data on microorganisms. This might partly stem from the phenomenon that microorganisms have generally higher dispersal rates compared to macroorganisms and are consequently considered to be less dispersal‐limited (Baas Becking and Nicolai 1934; Finlay 2002; Foissner 2006). Nevertheless, their roles as primary producers, decomposers and links in energy transfer to higher trophic levels underscore their importance in food webs and highlight the need for further research on their sensitivity to connectivity loss. Existing experimental studies on spatial connectivity between aquatic habitats, however, have generally targeted single trophic levels. In these studies, organisms representing higher trophic levels, such as zooplankton, were typically investigated in mesocosms (Gianuca et al. 2017; Howeth and Leibold 2010, 2013; Sinclair and Arnott 2018; Thompson et al. 2023; Thompson and Shurin 2012), apart from a few exceptions from microcosm systems (Cadotte and Fukami 2005; Carrara et al. 2012, 2014). In contrast, organism groups at lower trophic levels (e.g., diatoms and other microalgae) were rather studied in laboratory microcosms (de Boer et al. 2014; Eggers, Eriksson, and Matthiessen 2012; Guelzow et al. 2017). Even though connectivity loss may manifest differently at different trophic levels (Haegeman and Loreau 2013), potentially affecting food web functioning and energy transfer (Hagen et al. 2012; Liao, Bearup, and Blasius 2017), there have been relatively few studies encompassing multiple trophic levels within the same experimental setup. Furthermore, these studies usually applied connectivity to an external regional species pool (Limberger et al. 2019; Turunen et al. 2018; Vad et al. 2023). Modeling more realistic networks and food webs, and starting from setups with within‐network connectivity (as in large‐scale terrestrial experiments starting with an undisturbed system) could provide setups more powerful to disentangle the direct and indirect effects of connectivity and fragmentation, and eliminate bias due to potential mass effects produced by the repeated flow of organisms from an external source.

While connectivity loss has received considerable attention in metacommunity ecology, distinguishing its direct role from that of total habitat loss remains challenging based on empirical studies alone. Therefore, in the current study, we aimed to track the process of habitat‐network fragmentation (i.e., the loss of connectivity between habitats) starting with an intact habitat network, and to investigate its direct effects on biodiversity in experimental ponds, by keeping habitat amount and abiotic environment constant. More specifically, we tested whether habitat fragmentation in the form of connectivity loss among habitats reduces the local and regional richness of aquatic microorganisms in a 4‐month long experiment. In this regard, we compared the responses of prokaryotes and microeukaryotes, expecting that prokaryotes would be less affected by fragmentation due to their smaller size (De Bie et al. 2012) and larger population size (Berninger, Finlay, and Kuuppo‐Leinikki 1991; De Bie et al. 2012; Fenchel 1988). We also explicitly tested whether the local richness of microorganisms might be affected by food web interactions, specifically the biomass of their grazers, zooplankton, and whether the observed relationship is modulated by fragmentation. Ultimately, we aimed to identify the drivers of survival probability, including the direct and indirect effect of fragmentation and the role of initial regional abundance.

## 2 Materials and Methods

### 2.1 Experimental Setup and Sampling

We created six metacommunities (M1–M6) in an outdoor experimental setup. Each metacommunity consisted of five artificial ponds (mesocosms), with a total of 30 mesocosms set up randomly on an unshaded gravel field. As mesocosms, we used 225‐L UV‐resistant and food‐safe PEHD (high‐density polyethylene) plastic barrels, filled up to a 200‐L experimental volume. They were covered with mosquito nets to exclude macroinvertebrates and prevent coarse organic material (e.g., leaves) from falling into the mesocosms.

The mesocosms were filled with tap water and allowed to stand for de‐chlorination by evaporation for 4 days. Then, each mesocosm was inoculated with the inoculum representing a pooled plankton community from 10 intermittent lowland pools and ponds in the Pannonian Ecoregion (Text S1). This inoculum was equally distributed among the mesocosms by replacing 21 L of water removed from each mesocosm. Thereby, we ensured that the initial communities were identical in all mesocosms, containing microbial communities representative of natural lowland ponds. Nutrient concentrations were adjusted to match those measured in natural ponds, which were used for inoculation (0.7 mg L^−1^ total phosphorus, with 2.1 mg L^−1^ total nitrogen concentrations; Boros et al. 2017; Vad et al. 2017).

**FIGURE 1:**
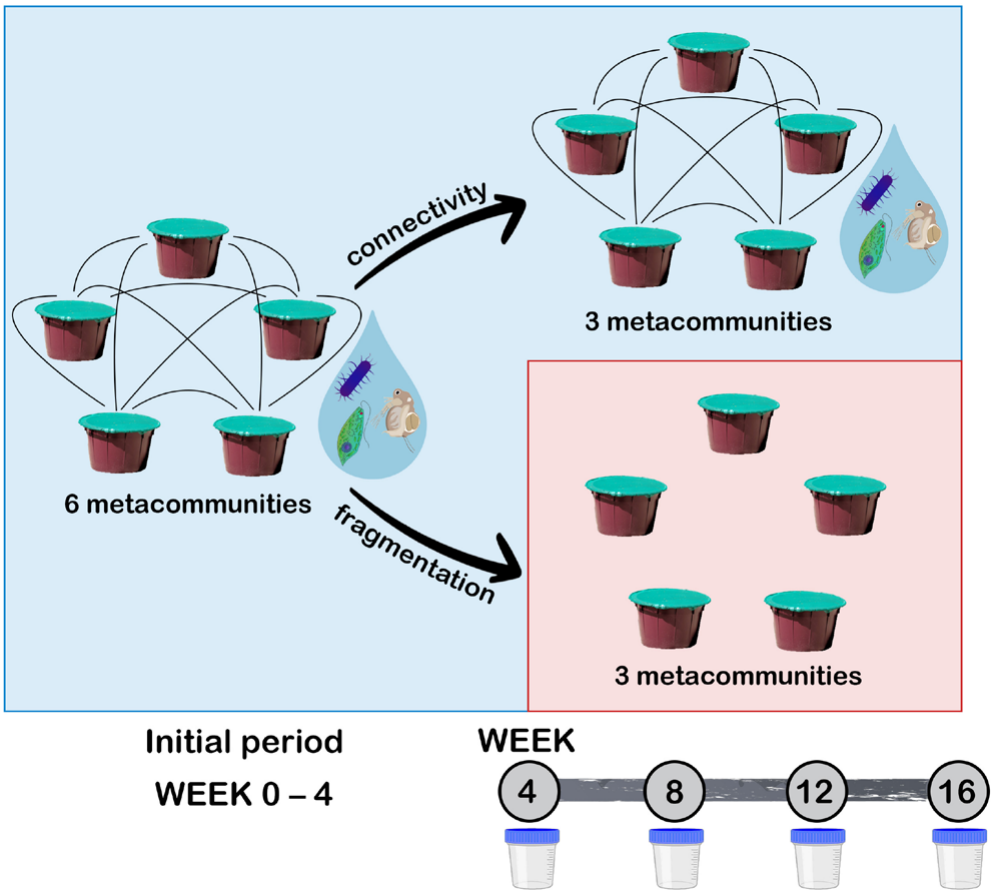
General setup of the experiment. Blue and red panels represent the two types of treatment (connectivity vs. fragmentation) applied for the metacommunities consisting of five habitats. Black solid lines between the habitats illustrate the weekly 1% water exchange. Sampling events are indicated by sample containers.

The experiment took place between 7 July and 27 October, 2020. Within each of the six metacommunities, dispersal treatment by 1% weekly water exchange was performed for 4 weeks (7 July–4 August; Figure 1). This was carried out by taking out 2.5 L of water with a transparent plexiglass tube plankton sampler from each mesocosm of a metacommunity after thoroughly homogenizing the water (stirring with a long stick). Then, the pooled water sample of the five mesocosms was redistributed into the same mesocosms thereby ensuring that 1% of the volume of each mesocosm, that is, 2 L was exchanged with the other four mesocosms of the metacommunity. After 4 weeks, we started the fragmentation treatment in three of the six metacommunities. To achieve this, we stopped the weekly dispersal treatment in three metacommunities (hereinafter referred to as “fragmented metacommunities,” M4–M6) to simulate connectivity loss while it was continued in the three control (hereinafter referred to as connected) metacommunities (M1–M3) for another 12 weeks (until 27 October; Figure 1). To ensure that the disturbance associated with the dispersal treatment did not cause any systematic differences between treatments, water in the fragmented mesocosms was also homogenized each week, however, without water exchange among mesocosms. Sampling equipment was always washed with tap water between mesocosms and experimental dispersal events to avoid cross‐contamination.

We sampled all mesocosms at the start of the fragmentation treatment and from that point, every 4 weeks resulting in four sampling events in total (Figure 1). To ensure a homogeneous distribution of plankton, each mesocosm was stirred prior to sampling. Then, to obtain samples of pro‐ and microeukaryote communities and water for nutrient analysis, a total of 1.5 L of water were taken out from three different locations (3 × 0.5 L) of each mesocosm using the same plexiglass tube sampler also used for dispersal. Water was first filtered through a 100‐μm mesh plankton net to remove most multicellular organisms. For the eDNA‐based analyses of pro‐ and microeukaryote communities, 250 mL of the filtered water was taken to the laboratory in a cooling box and filtered through an MF‐Millipore membrane filter (Merck, 25 mm Ø, 0.1 μm pore size) to retain biomass for DNA extraction. The filtered volume depended on the actual microbial biomass as we filtered water until filters were clogged or we reached 250 mL of total volume. The filters were then stored at −20°C until processing.

To track phytoplankton dynamics, we used chlorophyll‐a fluorescence (maximum fluorescence yield, Chl‐a, a.u.) as a proxy of total phytoplankton biomass, which was measured with a handheld fluorometer (AquaPen AP 110‐C, Photon System Instruments) at each of the four sampling events. Additional samples for the microscopic analysis of phytoplankton communities were taken during the last sampling event (Week 16). In total, 50 mL aliquots of the same 1.5 L representative sample that we collected for the eDNA‐based analyses of pro‐ and microeukaryote communities and nutrient analysis were fixed with 50 μL Lugol's iodine and stored at 5°C until subsequent analysis. Zooplankton sampling was also carried out at each sampling event. To do so, a total of 4 L of water were collected with the plexiglass tube sampler from three randomly chosen spots (1.5 L + 1.5 L + 1 L) of each mesocosm after thorough mixing. Thereafter, the pooled water was filtered through a 30‐μm mesh plankton net, and zooplankton samples were preserved in 70% ethanol. In parallel, abiotic environmental variables such as temperature, pH, electric conductivity, total phosphorus, and total nitrogen were also analyzed (Table S1). For a more detailed description of sample processing of phytoplankton, zooplankton, measurement of chlorophyll‐a fluorescence, and determination of physical and chemical variables, see Text S2.

From Week 8, filamentous green algae appeared at the water surface in the mesocosms. To account for their potential effect on the microbial communities (as competitors) and possible treatment‐specific differences in their presence and quantities, we also sampled them in Weeks 8, 12, and 16 (Table S2). Their sampling was carried out from the same 1.5 L water sample that we collected for the molecular samples. Filamentous algae that were present in this sample were retained on a 100‐μm mesh plankton net, immediately frozen and their dry weight after freeze‐drying was determined later in the lab (referred to as filamentous algae biomass—FB, g L^−1^). To test for treatment‐specific differences in filamentous algae biomass, analysis of variance (ANOVA) was performed in Weeks 8, 12, and 16, where treatment was involved as a fixed, and metacommunity ID as a nested factor (i.e., nested within the treatment). Filamentous algae persisted throughout the second half of the experiment, likely due to elevated summer temperatures combined with eutrophic conditions (Gladyshev and Gubelit 2019; Guo et al. 2022; Irfanullah and Moss 2005; Trochine et al. 2011; Zhu et al. 2020). However, their biomass did not show a treatment‐specific difference in any of the sampling campaigns (Table S2); therefore, we did not include it as an explanatory variable in the statistical analyses used for exploring microbial metacommunity patterns.

### 2.2 DNA Isolation, Amplification, Sequencing, and Data Processing

DNA extraction from the material retained on the membrane filters was carried out using the DNeasy PowerSoil Kit (Qiagen, Germany). The isolated DNA was amplified through polymerase chain reaction (PCR) to increase the quantity of fragments of taxonomic marker genes encoding 16S and 18S ribosomal RNA. The PCR products were then sent to Genomics Core Facility RTSF, Michigan State University, USA, for sequencing on an Illumina MiSeq platform (Illumina, USA). Raw sequencing reads were processed with mothur v.1.47.0 (Schloss et al. 2009). For further information on the isolation, amplification, sequencing, and data processing procedures, refer to Text S3.

### 2.3 Statistical Analysis

To track whether patch homogeneity lasted throughout the experiment, we tested for treatment‐specific differences in environmental variables, and the taxon richness (^α^S) and biomass (ZB, μg L^−1^) of zooplankton (as grazers) that might have affected the microbial metacommunities. In the case of environmental variables, permutational multivariate analysis of variance (PERMANOVA, permutations = 2000) was performed based on Euclidean distance of z‐score standardized data using the nested.npmanova() function in the “BiodiversityR” v. 2.15–4 package (Kindt and Coe 2005). ^α^S and ZB were analyzed using ANOVA at each of the four sampling events. In each PERMANOVA and ANOVA, treatment was considered as a fixed and metacommunity ID as a nested factor. As we found no systematic treatment‐specific difference in the environmental variables (Table S3) that could have introduced bias via environmental heterogeneity, we omitted using them in the analytical framework used for microbial metacommunity patterns. Zooplankton communities were exclusively composed of Rotifera and Cladocera taxa over the experiment (Figure S1, Table S4). A significant treatment effect on ZB emerged in Week 16 (Table S5); hence, we included it as an explanatory variable in the further data analyses to explore potential causal relationships with the microbial communities.

To obtain prokaryote and microeukaryote community datasets, we rarefied both 16S and 18S ASV sets separately to the read number of the sample having the lowest sequence number (13,835 reads for the 16S set and 2224 reads for the 18S set). All statistical analyses were carried out on the rarefied 16S (hereinafter referred to as prokaryotes) and 18S (microeukaryotes) community datasets separately using the R (v. 4.2.1) programming language (R Core Team 2022). Raw sequence reads are available in the European Nucleotide Archive (https://www.ebi.ac.uk/ena/browser/view/PRJEB78363) under reference number PRJEB78363 (Szabó et al. 2024), and the ASV sets with the related list of taxa in Tables [Link], [Link].

For each sampling event (Weeks 4, 8, 12, and 16), separate ANOVAs were run to test the possible effect of treatment (connectivity vs. fragmentation) on the number of observed ASVs (^α^S, i.e., local richness), the effective number of ASVs of PIE (^α^S_PIE_) and Pielou's evenness (J, referred to as “evenness” later on, as a measure of how evenly the number of individuals are distributed among the ASVs) at α‐scale, and the Whittaker's β‐diversity (β_S_ = ^γ^S/^α^S, i.e., compositional variation) in each metacommunity. In contrast to ^α^S being sensitive to changes in the number of rare species (ASVs in our case), ^α^S_PIE_ rather indicates the changes in the number of abundant species (McGlinn et al. 2019). In the models, treatment was included as a fixed and metacommunity ID as a nested factor. For the ANOVAs conducted on the last sampling event data, we also calculated partial η^2^ to assess effect sizes, given the significant treatment effects observed. The calculations were performed using the “effectsize” v. 0.8.9 package (Ben‐Shachar, Lüdecke, and Makowski 2020). To compare the number of observed ASVs at γ‐scale (^γ^S, i.e., regional richness) and the extrapolated richness across connected and fragmented metacommunities at each sampling event, sample‐size‐based rarefaction and extrapolation approach (Chao et al. 2014) using “iNEXT” v. 3.0.0 package (Hsieh, Ma, and Chao 2022) was applied. The 95% confidence intervals were calculated based on 10 bootstrap replications. To test whether treatment had a significant effect on ^α^S over the entire experimental period, generalized additive models (GAMs) were run with treatment as the main linear predictor, adding time (i.e., the week of sampling) with varying shapes of smooth according to individual metacommunities (*k* = 3 and *k* = 4 for prokaryotes and microeukaryotes, respectively). The models were built using the gam() function in the “mgcv” v. 1.8‐42 package (Wood 2017). Model diagnostics (including selection of *k* values) were inspected with the appraise() function of “gratia” v. 0.8.1 package (Simpson 2023).

To assess the possible treatment effect on Chl‐a over the whole experiment, and phytoplankton diversity at the last sampling event (Week 16), nested ANOVAs were performed on Chl‐a, and ^α^S, ^α^S_PIE_, J, and β_S_ of phytoplankton the same way as carried out for prokaryotes and microeukaryotes. Relative abundance (%) of groups of phytoplankton taxa per metacommunity was illustrated with stacked barplots. To identify taxa significantly associated with either treatment, an indicator species analysis was performed using the “indicspecies” v. 1.7.15 package (De Cáceres and Legendre 2009). Cell count data (cells mL^−1^) of phytoplankton taxa and assigned groups are presented in Table S10.

Linear mixed‐effects models (LMMs) using the “lme4” v. 1.1–35.1 package (Bates et al. 2015) were built to explore the potential effect of the treatment and ZB on ^α^S, ^α^S_PIE_, and J in prokaryotes and microeukaryotes in Week 16. In the models, treatment and ZB were included as fixed, while metacommunity ID as a nested random factor. In each case, we built two models, one without and another with the interaction of treatment and ZB, then compared them with a chi‐square test using the anova() function to select the best fit model. The interaction term only improved the model built for ^α^S in microeukaryotes (*χ* ^2^(1, −15.619) = 8.323; *p* < 0.01); therefore, we only kept it in this case.

To assess the relationships (direct and indirect effects) between treatment, ZB, and diversity (^α^S or J) in prokaryotes and microeukaryotes in Week 16, structural equation models (SEMs) were built. Here, we chose to only include ^α^S and not ^α^S_PIE_ given that they both describe local taxonomic richness (as opposed to evenness which entails additional information on dominance patterns). To find the best fitting SEM that describes the processes, we started by building simple SEMs considering both potential causal directions linking zooplankton and microbes (Figures S2 and S3). Each SEM consisted of linear mixed‐effects models (LMMs), where treatment was included as a dummy variable, and metacommunity ID as a random factor nested within treatment. Bootstrapping (1000 randomizations) was applied to determine the significance of standardized path coefficients. The models were compared and selected based on *R* ^2^ and Akaike information criterion (AIC) values. In microeukaryotes, ^α^S was better predicted when the LMM only included treatment as an explanatory variable (Figure S3) and not both treatment and ZB (Figure S2). Contrarily, J was better predicted by the LMM that included both treatment and ZB as explanatory variables (Figure S2). Accordingly, we built a final complex SEM (using pathways with the highest predictive values in the initial models) where the causal pathway assumed an indirect effect of microeukaryote ^α^S on microeukaryote J via ZB, and where the potential treatment effect was also included as a direct effect. In the case of prokaryotes, the simple SEMs only revealed a significant positive effect of connectivity treatment on ZB, but ^α^S, ^α^S_PIE_, and J were not significantly affected. Consequently, we did not build a complex SEM afterward. The results of each initial SEM are presented in the Supporting Information (Figures S2 and S3, Tables S11 and S12). SEMs were implemented using the “semEff” v. 0.6.1 package (Murphy 2022).

The effect of initial mean regional abundance (RA; i.e., mean abundance of the given ASV within a metacommunity in Week 4), ZB in Week 16, and the fragmentation treatment on the survival probability of the individual prokaryote and microeukaryote ASVs over the entire experimental period (i.e., presence or absence of the given ASV in Week 16) was studied with generalized linear mixed‐effects models (GLMMs). These GLMMs were built with binomial function, where RA, ZB, and treatment were included as fixed, while metacommunity ID as a nested random factor. Both for prokaryotes and microeukaryotes, we compared a model without and including the interaction between RA and treatment as well as between ZB and treatment with chi‐square test using the anova() function. As interaction terms significantly improved the model fits (prokaryotes: *χ* ^2^(2, 31,808) = 25.099, *p* < 0.001; microeukaryotes: *χ* ^2^(2, 35,550) = 7.820, *p* < 0.05), we retained both of them in both models. RA and ZB were z‐score standardized to eliminate the bias that can arise from the different measurement units and scales. Odds ratios and 95% confidence intervals were calculated using the tab_model() function of “sjPlot” v. 2.8.15 package (Lüdecke 2023).

In the main part of the paper, we present the results obtained for prokaryotes and microeukaryotes at the last sampling event. All other results are provided in the Supporting Information. Although whisker plots and scatter plots were created based on raw datasets for an easier visual comparison of different organism groups and sampling events, when necessary, dependent variables were transformed to achieve normal distribution. Applied transformations are presented in the relevant tables along with the results of statistical tests (Tables S2, S5, S11–S15, S17 and S18).

## 3 Results

**FIGURE 2:**
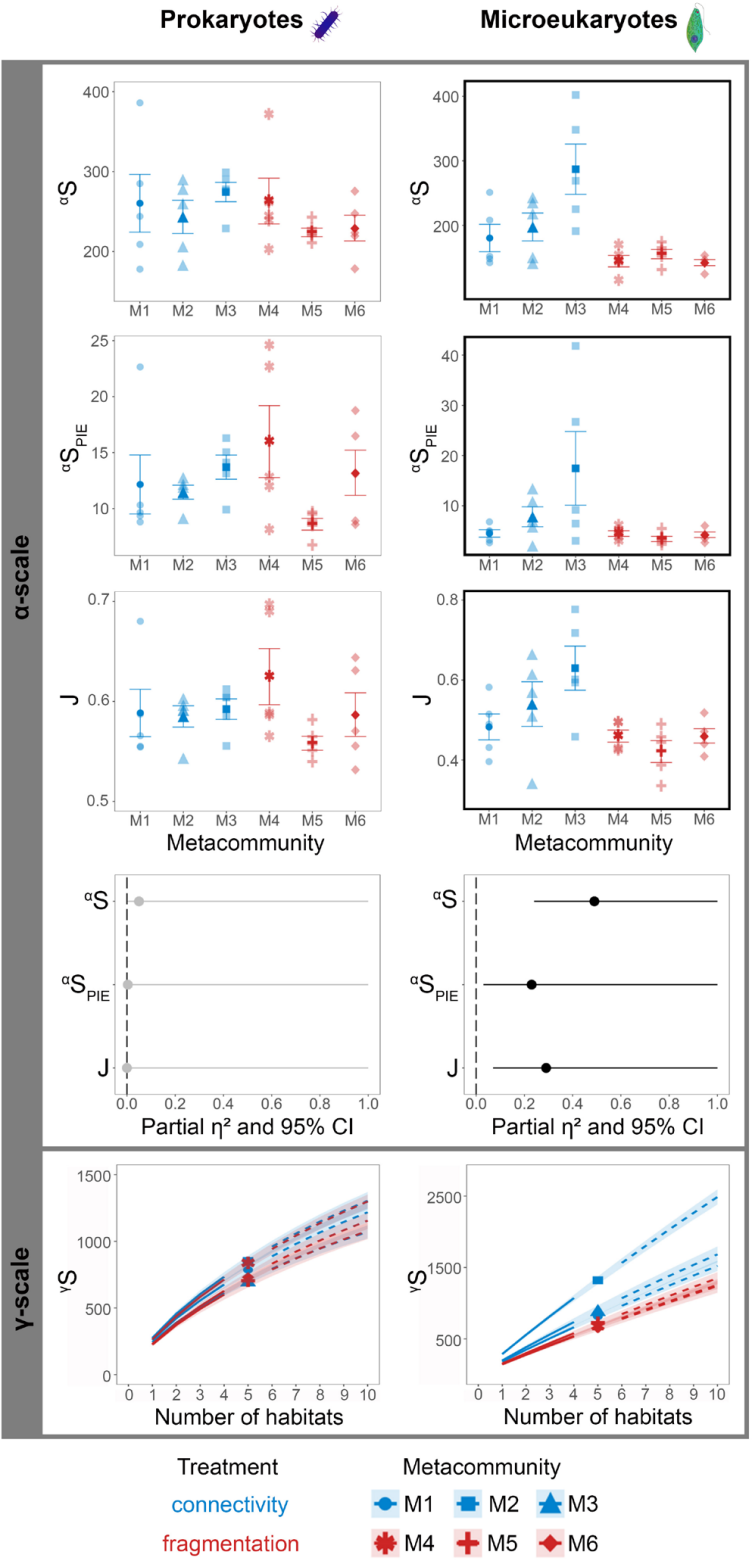
Number of observed ASVs (^α^S), effective number of ASVs of PIE (^α^S_PIE_) and evenness (J) at α‐scale in prokaryotes and microeukaryotes in each metacommunity at the end of the experiment (Week 16), along with partial *η* ^2^ and 95% confidence intervals for each ANOVA (black circles with horizontal whiskers: significant effect; gray circles with horizontal whiskers: non‐significant effect). Accumulation curves with the number of observed ASVs (^γ^S) at γ‐scale, along with extrapolated estimates (dashed lines) and 95% confidence intervals (error bands) are also displayed. Boxes marked with a bold black frame indicate a significant treatment effect (*p* < 0.05) resulting from the ANOVA (see Table S13 for the statistical summary).

Local richness of microeukaryotes, that is, both ^α^S and ^α^S_PIE_ were significantly lower in the fragmented metacommunities compared to the connected ones at the end of the experiment (Week 16; Figure 2, Table S13). In prokaryotes, a significant negative effect of fragmentation on ^α^S emerged by Week 12, while it disappeared by Week 16 with no significant effect on ^α^S and ^α^S_PIE_. Fragmentation resulted in a significantly lower J in microeukaryotes by the end of the experiment (Figure 2, Table S13). In contrast, prokaryote J was not affected by treatment. The largest effect size (partial *η* ^2^) was obtained for microeukaryote ^α^S, followed by J, and then ^α^S_PIE_ indicating that treatment accounted for the highest proportion of variance in ^α^S (Figure 2). When analyzing the temporal pattern of ^α^S over the entire experimental period, we also found a negative effect of fragmentation on microeukaryotes (GAM Estimate = −0.103, *t* = −2.601, *p* < 0.05) but no effect on prokaryotes (GAM Estimate = −11.831, *t* = −1.534, *p* = 0.128).

We did not find a significant treatment effect on β_S_ in microeukaryotes (Table S13). In prokaryotes, β_S_ was significantly lower in the connected metacommunities in Week 12, but even this difference disappeared by the end of the experiment (Table S13).

At the regional scale (γ), fragmentation resulted in a lower number of ASVs in microeukaryotes by the end of the experiment. This was evidenced by the accumulation curves and no overlap between the 95% confidence intervals between treatments (Figure 2, Figure S4). In prokaryotes, no treatment effect on ^γ^S was found for the entire experimental duration (Figure 2, Figure S4).

Chl‐a was not affected significantly by the treatment (Table S14). ^α^S_PIE_ of phytoplankton (based on microscopic identification) was significantly decreased by the fragmentation treatment at the end of the experiment, while we found no effect on ^α^S, J, and β_S_ (Table S15), similarly to the results obtained for the microeukaryote communities based on metabarcoding data. Picophytoplankton was a dominant member of phytoplankton under fragmentation treatment (Figure S5), while this group was significantly reduced in the connected metacommunities based on the indicator species analysis (Table S16). The relative proportion of unicellular chlorophytes showed the opposite pattern, with a significant association with the connectivity treatment (Figure S5, Table S16).

**FIGURE 3:**
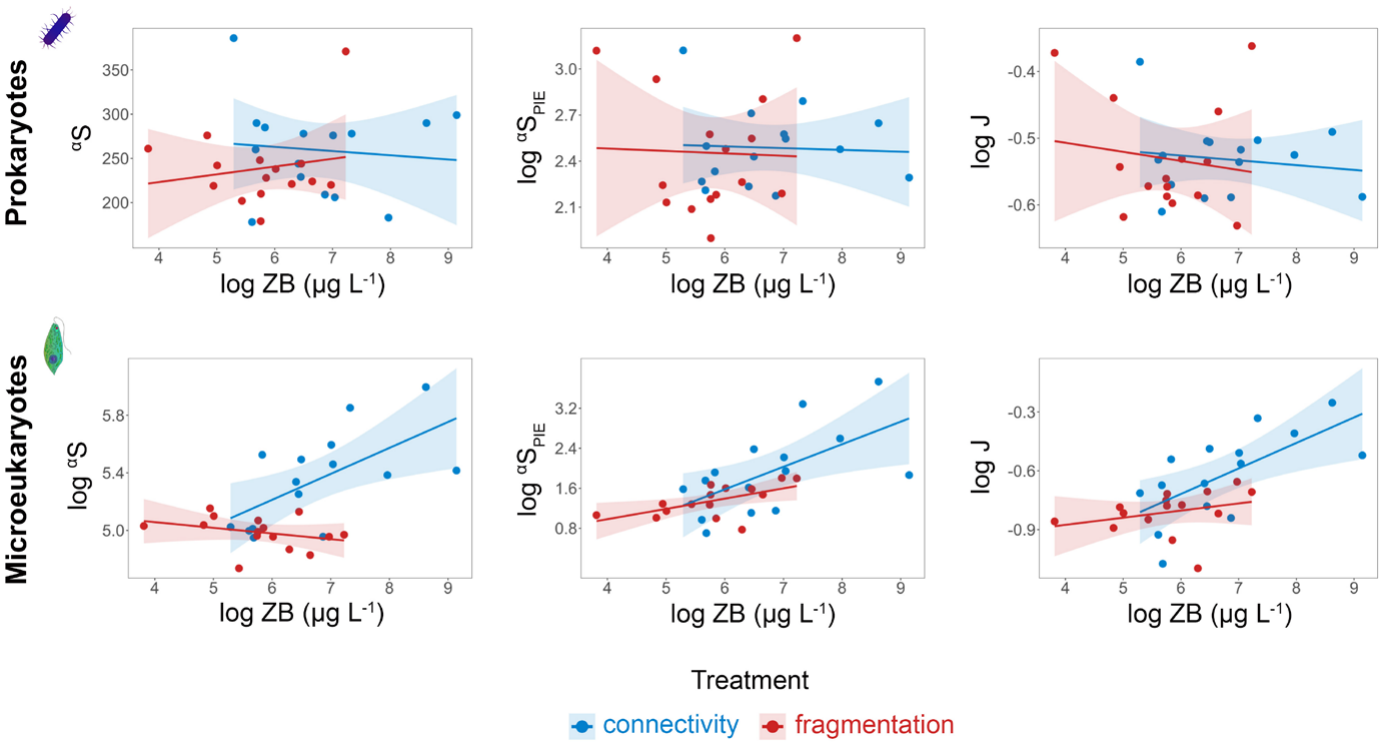
Linear regression plots illustrating the change in the number of observed ASVs (^α^S), effective number of ASVs of PIE (^α^S_PIE_), and evenness (J) as a function of zooplankton biomass (ZB) in the two treatments (connectivity and fragmentation) for prokaryotes and microeukaryotes at the end of the experiment (Week 16). Solid lines represent the fitted linear models (error bands: standard errors). ZB, ^α^S_PIE_, and J were log‐transformed in both cases, and ^α^S in the case of microeukaryotes. Summary statistics of the LMMs, including metacommunity ID as a random factor are presented in the Supporting Information (Table S17).

Prokaryote ^α^S, ^α^S_PIE_, and J were not affected significantly by either treatment or ZB at the end of the experiment (Week 16) based on the LMM and SEM (Figure 3, Figure S2, Tables S11 and S17). In contrast, ^α^S of microeukaryotes had a positive relationship with ZB in the connected metacommunities and a negative relationship in the fragmented ones (Figure 3, Table S17; ANOVA: *P*_Treatment*log(ZB)_ < 0.05, *F*_Treatment*log(ZB)_ = 7.144). Microeukaryote ^α^S_PIE_ and J showed an increase with the increase of ZB irrespective of treatment (Figure 3, Table S17).

**FIGURE 4:**
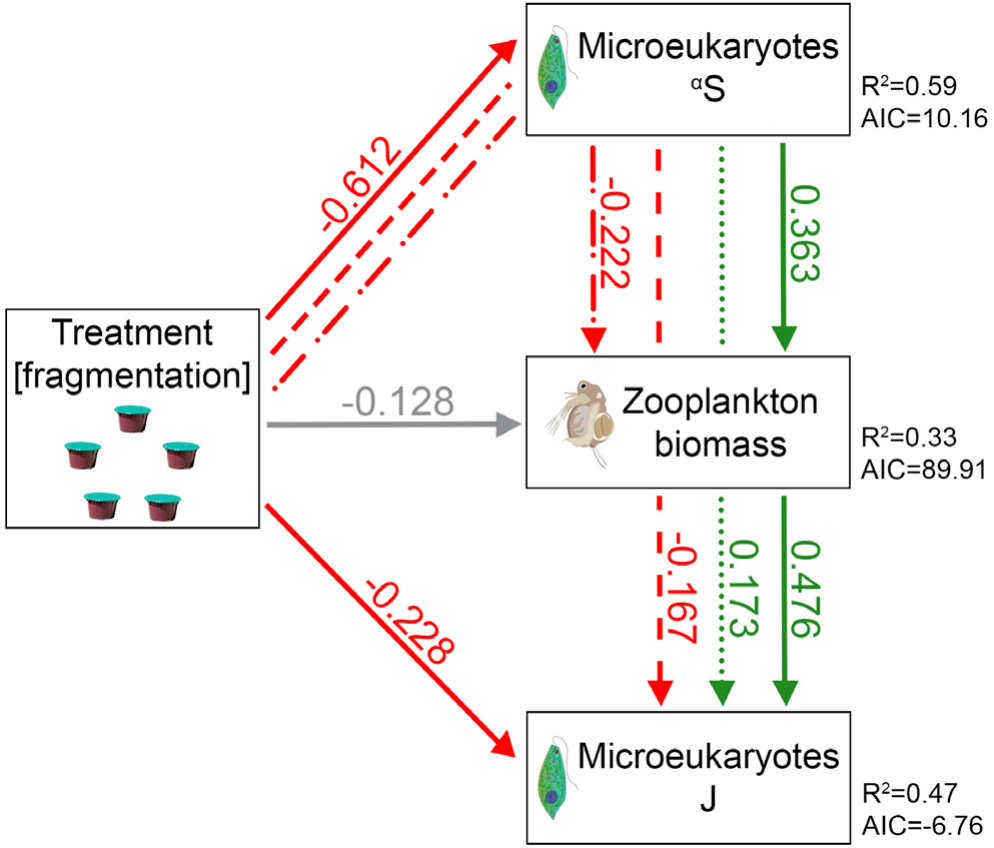
Structural equation model (SEM) showing the relationships (direct and indirect effects) between treatment, zooplankton biomass, and diversity (^α^S or J) in microeukaryotes at the end of the experiment (Week 16). Variables are represented by boxes and the directional relationships by single‐head arrows (solid line: direct effect; dashed, dotted, and dash‐dotted lines: indirect effects; green: significant positive effect; red: significant negative effect; gray: non‐significant effect). Standardized path coefficients are shown on the arrows. *R* ^2^ and AIC values for the component models are indicated next to the boxes of endogenous variables. ^α^S, zooplankton biomass, and J were log‐transformed prior to the analysis. In the models, microeukaryote ^α^S, zooplankton biomass, and treatment were included as fixed, and metacommunity ID as a random factor nested within treatment (see Table S18 for the statistical summary).

Based on the SEM (Figure 4, Table S18), fragmentation had a significant negative direct effect on microeukaryote ^α^S, while the direct effect of microeukaryote ^α^S on ZB, and of ZB on microeukaryote J was a significant positive effect. Although treatment directly did not increase ZB significantly, its indirect negative effect including microeukaryote ^α^S as a mediator was significant, which is in line with the negative relationship between microeukaryote ^α^S and ZB under fragmentation (Figure 3). Microeukaryote J significantly decreased as a response to fragmentation, and in this case, the direct treatment effect was stronger than the indirect (i.e., the effect of treatment on microeukaryote J with the mediation of microeukaryote ^α^S and ZB) (Figure 4, Table S18).

**FIGURE 5:**
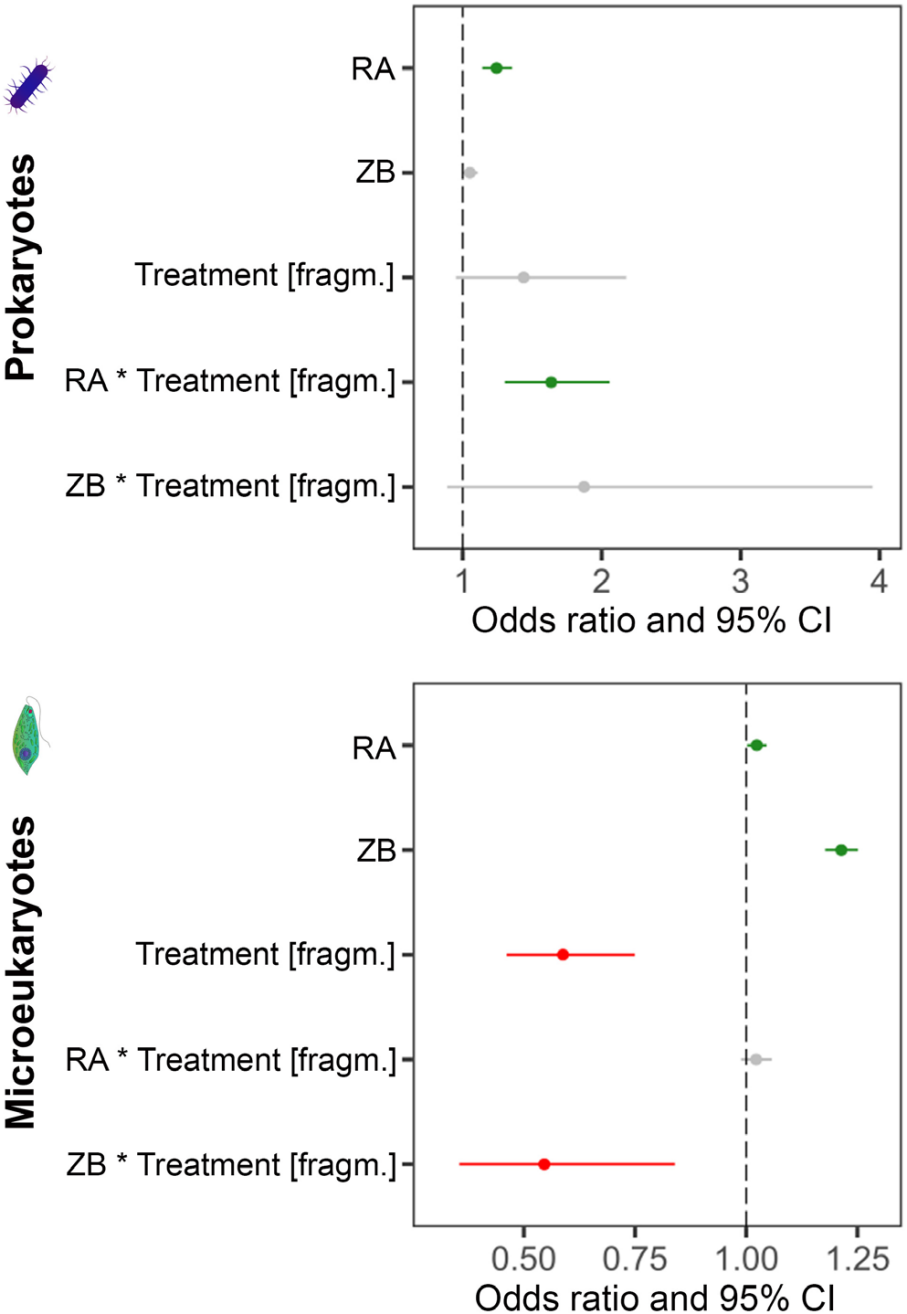
Forest plots demonstrating the effect of the initial mean regional abundance (RA), zooplankton biomass (ZB) in Week 16, treatment, and the interaction of treatment with RA and ZB on the survival probability of individual ASVs by the end of the experiment (red: significant negative, green: significant positive effect; gray: non‐significant effect). The plots are based on odds ratios (closed circles, with whiskers representing 95% confidence intervals) in the generalized linear mixed‐effects models (GLMMs) performed on the presence/absence of the ASVs in Week 16 in prokaryotes and microeukaryotes. In the models, RA, ZB, and treatment were included as fixed, and metacommunity ID as a random factor nested within treatment.

The initial mean regional abundance significantly affected the survival probability of both prokaryotes and microeukaryotes (based on GLMM with an odds ratio > 1). Besides the regional abundance, ZB in Week 16, treatment and also their interaction significantly affected the survival probability of microeukaryote ASVs. These results indicate that ZB had different effects in the two treatments: an increase in ZB increased the survival probability in microeukaryotes in the connected metacommunities while decreasing it in the fragmented ones (Figure 5). In prokaryotes, the treatment did not affect the survival probability; however, the interaction effect of regional abundance and treatment was significant. This indicates that an increase in the regional abundance increased the survival probability in the connected metacommunities to a lesser extent than in the fragmented metacommunities (Figure 5).

## 4 Discussion

Although several empirical studies have addressed the potential impact of habitat fragmentation (e.g., Branco et al. 2012; Hamer 2016; Hamer, Mechura, and Puky 2023; Johnson et al. 2013; Rösch et al. 2013), distinguishing the direct effects of connectivity loss from those of habitat loss remains challenging in these studies without controlling for many underlying factors, such as changes in habitat quality or quantity. Here, we provided clear experimental evidence on the negative effects of habitat fragmentation, specifically its connectivity loss component, on local and regional biodiversity. In our study based on experimental pond networks, we found that without changes in underlying local environmental conditions or loss of total habitat amount, connectivity loss alone exerted a strong negative effect on both local and regional richness and evenness of unicellular microeukaryotes. This negative effect could be observed both in ^α^S and ^α^S_PIE_, indicating that rare and abundant taxa were all affected. In line with our expectation, diversity of prokaryotes was less affected, although the initial mean regional abundance of prokaryote taxa significantly affected their survival probability, indicating a potential extinction debt. We also found that the negative effect of connectivity loss was partly related to the dispersal‐mediated trophic interactions with grazers. Overall, our study clearly demonstrates that loss of connectivity within a pond network can lead to a significant loss of microbial biodiversity.

Given the absence of environmental gradients, our experimental metacommunity can be considered as representing a case where patch dynamics is the major process of community assembly. In such metacommunities, interspecific interactions play a predominant role in community assembly modulated by dispersal as a key process (Leibold et al. 2004; Thompson et al. 2020). In our study, we expected a direct effect of habitat fragmentation in the form of connectivity loss especially in the microeukaryote metacommunities. Indeed, by the end of the 16‐week experiment, we found significantly lower local richness of microeukaryotes in the fragmented metacommunities compared to the connected ones. This was apparent both in ^α^S and ^α^S_PIE_, indicating a negative effect on both rare and abundant taxa. A similar difference also emerged at the regional scale, resulting in lower regional diversity. Overall, these results clearly point out that the disruption of weekly dispersal was able to lead to the loss of several taxa. These findings were further supported by our analysis on direct and indirect effects and also by a separate analysis based on survival probabilities, providing further evidence for the negative effect of connectivity loss on the survival of microeukaryotes during the experiment.

Besides local richness, evenness of the microeukaryote communities under connectivity loss was also significantly lower compared to those where connectivity was maintained until the end of the experiment. Our results support that connectivity can alter the outcome of interspecific interactions by facilitating immigration of species from neighboring habitats, thereby decreasing the local dominance of competitively superior species (Leibold et al. 2004). Habitats affected by connectivity loss may exhibit reduced evenness as competitively inferior species may get outcompeted due to the reduced efficiency of rescue effects via immigration (Brown and Kodric‐Brown 1977; Dey and Joshi 2013; Eriksson et al. 2014; Marini et al. 2014). Furthermore, local survival probabilities can strongly depend on initial differences in population size (Kim et al. 2016; Matthies et al. 2004), as it was also supported by our analyses. Given that initial mean regional abundance was an important predictor of the survival of microeukaryotes in both treatments (connectivity vs. fragmentation), this might also point at the role of priority effects to some extent. These strong dominance patterns were enhanced by both the direct and indirect effects (by decreasing the biomass of zooplankton grazers via reducing microeukaryote richness) of connectivity loss and in the fragmentation treatment.

Besides its effect on the local scale, habitat fragmentation also led to significantly lower regional richness compared to those metacommunities where continuous connectivity ensured the dispersal of organisms evidenced by the species accumulation curves. This was in agreement with our prediction based on earlier theoretical (Thompson, Rayfield, and Gonzalez 2017) and experimental studies (Gilbert, Gonzalez, and Evans‐Freke 1998). The effects of connectivity loss on regional biodiversity can be less straightforward in more complex natural settings, involving heterogeneous habitat patches and complex food webs (e.g., Evans et al. 2017; Gibb and Hochuli 2002; Gibson et al. 2013). Our results, however, suggested the loss in regional diversity under weakened rescue effects due to the disruption of the dispersal network.

While homogeneity of the abiotic environment and phytoplankton biomass (approximated by Chl‐a fluorescence) persisted during the experiment, indicated by no significant differences among treatments, fragmentation reduced ZB by the end of the experiment. This effect on ZB was not coupled with a parallel effect on zooplankton taxon richness (Table S5), and it indicates potential differences in the biotic interactions between the treatments. A general negative effect of fragmentation on both the diversity of microeukaryotes and the biomass of their zooplankton grazers may be a result (and actually a combination) of three processes. First, connectivity loss may have a direct negative effect on both microeukaryotes and zooplankton. A direct effect seems to be the case for microeukaryotes, supported by a series of complementary analyses of both local and regional diversity, including the survival probability analysis showing both the importance of initial mean regional abundances and the effect of connectivity loss. At the same time, the negative effect of fragmentation on ZB was more of an indirect effect (Figure 4). The lack of direct significant effect on zooplankton taxon richness could have been also linked to differences in taxonomic resolution used for the two groups (metabarcoding: a total of 11,134 ASVs for microeukaryotes; taxon richness via microscopic analysis for zooplankton: 16 taxa). However, the standard microscopic analysis of phytoplankton (which was more comparable to the microscopic analysis of zooplankton in terms of taxonomic resolution, with a total of 82 taxa encountered during the experiment) largely corroborated the metabarcoding‐based results and led us to the same conclusion. These results therefore rather indicate potentially different trophic relationships under fragmentation.

Second, phytoplankton biomass (Chl‐a fluorescence; Table S15) did not differ across the treatments; hence, the quantity of algal food for zooplankton was presumably similar in both treatments. In contrast to the fragmented metacommunities, maintaining connectivity in the connectivity treatment could have indirectly contributed to higher ZB via an increased diversity of their food resource (Striebel et al. 2012; Marzetz et al. 2017). This was indeed supported by the results of our complex structural equation model, where fragmentation had both a significant direct effect on microeukaryote ^α^S and no direct, but an indirect effect on ZB via mediation of local diversity in microeukaryotes.

Third and finally, changes in the biotic interactions between zooplankton grazers and microeukaryotes might have also contributed to the changes in local diversity patterns via another indirect pathway. Our results suggest that the lower evenness of microeukaryotes under fragmentation resulted both from the direct negative effect of the treatment and the effect of a lower ZB. In the connectivity treatment, the higher biomass of the relatively small‐sized cladocerans (*Moina* and *Macrothrix*), being dominant in the communities, seemingly exerted an overall positive grazing effect on microeukaryotes. Intermediate levels of grazing can be considered as a form of intermediate disturbance (Colburn 2008; Joubert, Pryke, and Samways 2017; Shea, Roxburgh, and Rauschert 2004; Yuan et al. 2016). It is possible that the dominant cladocerans exerted such an effect, which was supported by the positive relationship of ZB with both microeukaryote diversity and evenness in the connectivity treatment (Figure 3). Zooplankton communities dominated by large‐bodied generalist cladocerans such as *Daphnia magna* might exert different effects on their prey communities, for example, even masking the positive effect of connectivity on local diversity (Berga et al. 2015). In contrast, the small‐sized cladocerans and rotifers in our experiment likely contributed to decreasing the dominance of a selected set of otherwise superior competitor species either via more selective grazing (based on size, taste, concentration, nutritional value, etc.; Sterner 1989) or by a general but intermediate grazing pressure that could benefit the competitively inferior species (Lubchenco 1978; Pulungan et al. 2019). The pattern we found that evenness might be linked to treatment‐specific differences in grazing effects was also confirmed by our microscopic analyses that showed a higher dominance of picophytoplankton in the fragmentation treatment (Table S10, Table S16, Figure S5). These small‐sized unicellular algae are within the filtration range of *Moina* (2–40 μm; Pagano 2008) and several rotifers (Pourriot 1977; Rothhaupt 1990); thus, the high biomass of these zooplankton taxa at the end of the experiment seemed to be linked to the decline of pico‐size algae in the connected mesocosms.

Both pro‐ and microeukaryotes are known to be efficient passive dispersers across long distances (Genitsaris, Moustaka‐Gouni, and Kormas 2011; Luef et al. 2007; Mony et al. 2020; Szabó et al. 2022), and their communities often show no or only a weak spatial structuring (Barta et al. 2024; Beisner et al. 2006; Padial et al. 2014). At the same time, there is also increasing evidence for the importance of connectivity for sustaining biodiversity and temporal stability of local communities in these groups (e.g., Berga et al. 2015; Engel, Matthiessen, and Eriksson 2020; Guelzow et al. 2017; Thompson, Beisner, and Gonzalez 2015; Vad et al. 2023). Among the two, prokaryotes have been considered to be less dispersal limited due to their smaller size (De Bie et al. 2012), their larger population sizes of a few orders of magnitude compared to microeukaryotes (Berninger, Finlay, and Kuuppo‐Leinikki 1991; De Bie et al. 2012; Fenchel 1988), and their higher rate of propagule production (i.e., shorter generation time) (Zubkov 2014). In line with these, we predicted a comparably smaller effect of fragmentation on prokaryotes than on microeukaryotes. This was supported by our results based on the analyses of both local and regional diversity and survival probabilities, with no significant direct effect of fragmentation on prokaryotes. On the other hand, mean regional abundance at the start of the fragmentation treatment was an even stronger predictor of local survival in prokaryotes than in microeukaryotes, especially when predicting survival in the fragmentation treatment. This might point at higher levels of monopolization in the absence of dispersal (Loeuille and Leibold 2008; Urban and De Meester 2009) and a potentially longer time frame that would have been necessary to detect changes in species richness. Larger populations or regionally more frequent species of a metacommunity are less prone to extinction driven by stochastic events (De Silva and Leimgruber 2019; Eriksson et al. 2014; Kim et al. 2016; Lande, Engen, and Sæther 2003; Matthies et al. 2004). ASVs with lower initial mean abundance might also experience slow declines over a longer time frame, eventually leading to extinction events also in prokaryotes. This delayed response might indicate a potential extinction debt in this group as a response to fragmentation.

An important novelty of our study lies in the way of application of the fragmentation treatment, which was independent of habitat amount reduction: We modeled connected sites that later got disconnected, thereby mimicking the natural dynamics of connectivity loss in a landscape. Furthermore, in contrast to several previous studies that applied a constant dispersal from an external regional species pool, we maintained a within‐network connectivity, thus avoiding bias due to the potential mass effects and a potential loss in species due to a “mesocosm effect”; this occurs when species are lost during the first phase of an experiment due to the recent inoculation from natural ponds, which somewhat differ in their local characteristics from the experimental conditions (see, e.g., Adey et al. 1996; Limberger et al. 2019; Williams 2002). Such effects would have made it more difficult to track treatment‐specific extinction events. Nevertheless, the results we found also indicate that longer time (even months) might be needed for a detectable signal of fragmentation in such an experimental setup.

Habitat fragmentation can negatively affect biodiversity and alter ecosystem functioning in several direct and indirect ways. Although relatively much is known about its effects on terrestrial (Gonzalez et al. 1998; Graham et al. 2022; Haddad et al. 2017) and aquatic macroorganisms (Hamer 2016; Hamer, Mechura, and Puky 2023; Johnson et al. 2013; Rösch et al. 2013), aquatic prokaryotes and microeukaryotes, most of which act as important producers or decomposers in the food web, are still understudied in this regard. Our experimental study demonstrates that connectivity loss, that is, disruption of dispersal among habitat patches plays a crucial role by directly reducing both local and regional richness as well as community evenness. Our results were especially explicit in unicellular microeukaryotes, which may be linked to their smaller population sizes (Berninger, Finlay, and Kuuppo‐Leinikki 1991; De Bie et al. 2012; Fenchel 1988) and longer generation times (Zubkov 2014) compared to prokaryotes. We also found strong evidence that connectivity loss can indirectly decrease microeukaryote evenness via modulating biotic interactions, more precisely, the strength of zooplankton grazing effect. Overall, these results highlight that maintaining connectivity in networks of such highly sensitive small waterbodies as ponds is essential to preserve microbial diversity and ecosystem functioning. Furthermore, we emphasize that for better understanding the direct and indirect processes resulting from connectivity loss, and thus being able to assist conservation strategies and management practices, longer‐term experiments incorporating complex food webs are required.

## Author Contributions

**Beáta Szabó:** conceptualization, data curation, formal analysis, investigation, methodology, visualization, writing – original draft. **Máté Váczy‐Földi:** conceptualization, data curation, formal analysis, investigation, methodology, visualization, writing – original draft. **Csaba F. Vad:** conceptualization, funding acquisition, investigation, methodology, supervision, writing – review and editing. **Károly Pálffy:** conceptualization, investigation, methodology, writing – review and editing. **Thu‐Hương Huỳnh:** investigation, writing – review and editing. **Péter Dobosy:** investigation, methodology, writing – review and editing. **Ádám Fierpasz:** investigation, writing – review and editing. **Zsuzsanna Márton:** data curation, investigation, methodology, writing – review and editing. **Tamás Felföldi:** methodology, writing – review and editing. **Zsófia Horváth:** conceptualization, data curation, funding acquisition, investigation, methodology, resources, supervision, writing – original draft.

## Conflicts of Interest

The authors declare no conflicts of interest.

## Supporting information


Data S1.

**Table S1.** Environmental variables measured in the mesocosms. Sampling event, mesocosm ID, metacommunity ID and treatment are indicated.

**Table S4.** Zooplankton taxa (ind L^−1^) identified in the mesocosms. Sampling event, mesocosm ID, metacommunity ID and treatment are indicated.

**Table S6.** 16S ASV set rarefied to 13,835 read per sample (referred to as prokaryotic dataset). Sampling event, mesocosm ID, metacommunity ID and treatment are indicated.

**Table S7.** 18S ASV set rarefied to 2224 read per sample (referred to as microeukaryotic dataset). Sampling event, mesocosm ID, metacommunity ID and treatment are indicated.

**Table S8.** List of taxa assigned to the 16S ASV set.

**Table S9.** List of taxa assigned to the 18S ASV set.

**Table S10.** Phytoplankton taxa (cells mL^−1^) identified in the mesocosms. Sampling event, mesocosm ID, metacommunity ID and treatment are indicated.

## Data Availability

The data that support the findings of this study are openly available in Dryad at https://doi.org/10.5061/dryad.fj6q5744j and the European Nucleotide Archive at https://www.ebi.ac.uk/ena/browser/view/PRJEB78363.
